# ICD‐Related Emergencies in Prehospital Care: A Descriptive Survey of Self‐Reported Experience and Equipment Availability Among Polish Paramedics

**DOI:** 10.1155/emmi/2737258

**Published:** 2026-05-28

**Authors:** Wojciech Telec, Tomasz Kłosiewicz, Magdalena Dimmich, Marta Szczygieł, Maria Krzemińska, Rafał Wójcik, Artur Baszko

**Affiliations:** ^1^ 2nd Cardiology Clinic, Poznan University of Medical Sciences, 194 28 czerwca 1596r. Street, Poznań, 61-701, Poland, ump.edu.pl; ^2^ Department of Medical Rescue, Poznan University of Medical Sciences, 7 Rokietnicka Street, Poznań, 60-608, Poland, ump.edu.pl; ^3^ The Student Scientific Society of Poznan University of Medical Sciences, 10 Fredry Street, Poznań, 61-701, Poland

**Keywords:** attitudes, continuing, defibrillators, education, emergency medical services, equipment and supplies, implantable, medical, practice

## Abstract

**Background:**

The number of patients with implantable cardioverter‐defibrillators (ICDs) is steadily increasing, making encounters with ICD‐related emergencies more frequent in prehospital care. Paramedics are often the first healthcare professionals to manage these situations; however, data on their exposure to ICD‐related emergencies and access to appropriate equipment remain limited. This study aimed to describe Polish paramedics’ self‐reported experience with ICD‐related emergencies and the availability of ICD deactivation magnets in prehospital settings.

**Methods:**

A descriptive cross‐sectional survey was conducted among Polish paramedics between April and November 2024 using an anonymous online questionnaire. The survey assessed demographic and professional characteristics, self‐reported exposure to ICD‐related emergencies, perceived familiarity with selected ICD‐related concepts, and access to ICD deactivation magnets. Data were analyzed descriptively, with exploratory subgroup analyses performed for selected variables.

**Results:**

A total of 340 paramedics participated in the survey. Most respondents reported having assisted patients experiencing ICD discharges, including during cardiopulmonary resuscitation. Self‐reported familiarity with ICD‐related terminology and ECG features varied across items. Only 7.93% of respondents reported access to an ICD deactivation magnet in their ambulance, most often self‐provided rather than officially supplied. Greater professional experience was associated with more frequently reported exposure to ICD‐related emergencies.

**Conclusions:**

Our study suggests that ICD‐related emergencies are commonly encountered in prehospital practice, while access to ICD deactivation magnets remains limited. The findings reflect self‐reported experience and perceived familiarity rather than objectively assessed competence and highlight potential system‐level considerations for prehospital emergency care.

## 1. Introduction

Implantable cardioverter‐defibrillators (ICDs) are the standard treatment for preventing sudden cardiac death in patients at high risk, including those with symptomatic heart failure and low left ventricular ejection fraction, as well as individuals with a history of life‐threatening ventricular arrhythmias [1].

The ongoing aging of the Polish population and advancements in cardiology have led to an increasing number of ICD implants. According to the European Society of Cardiology, Poland currently has an implantation rate exceeding 200 per million people annually [2]. As the number of patients with ICDs rises, paramedics are more frequently encountering them in emergency settings. These patients represent a diverse group, including individuals with ischemic heart disease, heart failure, cardiomyopathies, and inherited channelopathies [3].

Effective prehospital management of ICD‐related complications is crucial, yet it requires paramedics to have a solid understanding of ICD function, placement, and potential complications. Key clinical scenarios include electrical storms (ESs), inappropriate shocks due to supraventricular arrhythmias or oversensing, and hardware malfunctions [2, 4, 5].

Despite ICDs being life‐saving devices, they can also present complications that require immediate intervention. The experience of receiving an ICD shock can cause significant distress, further complicating emergency care [6].

In addition, magnets can be used to temporarily deactivate ICD therapy in cases of inappropriate shocks, ES, or device malfunction. The magnet can be used in patients with ES, especially when the patient experiences recurrent shocks due to hemodynamically well‐tolerated ventricular tachycardia (VT), inappropriate shocks due to supraventricular arrhythmia, and oversensing or suspected hardware malfunction [7–10].

Moreover, in case of cardiac arrest with a shockable rhythm and ineffective treatment from ICD, it is recommended to use a magnet to disable the device and place pads at least 8 cm from the device or use alternative pad placements (anterior–lateral or anterior–posterior) [7, 11].

However, despite the effectiveness of this simple intervention, ICD deactivation magnets remain unavailable in many Polish ambulances.

This study aimed to describe Polish paramedics’ self‐reported experience with ICD‐related emergencies and the availability of ICD deactivation magnets in prehospital settings.

## 2. Methods

### 2.1. Legal Issues

According to Polish law and the principles of Good Clinical Practice, survey studies do not meet the criteria of a medical experiment. Therefore, obtaining approval from the bioethics committee is generally not required. The survey was conducted anonymously using Google Forms. No personally identifiable data were collected. IP addresses were not stored, and responses were accessed only by the research team. Data were processed in compliance with the General Data Protection Regulation.

### 2.2. Study Protocol and Recruitment

The study utilized a survey designed by the authors and developed using Google Forms. The survey was distributed among paramedics between April and November 2024.

Participants were recruited using a nonprobability convenience sampling strategy. The survey link was distributed via direct email invitations within professional paramedic networks and presented to registered paramedics during a national scientific conference using a QR code, allowing participants to complete the questionnaire on their personal mobile devices. Participation was voluntary and anonymous. The survey used an open‐link design without individualized invitations; no additional tool was used to prevent multiple entries; therefore, duplicate entries cannot be excluded. The study was prepared and reported in accordance with the STROBE and CROSS guidelines, and the corresponding checklists are available as Supporting Information (Supporting Information 1: STROBE checklist; Supporting Information 2: CROSS checklist).

### 2.3. Outcomes and Questionnaire

The primary outcome of the study was the self‐reported exposure to ICD‐related emergencies and access to ICD deactivation magnets. Secondary outcomes included self‐reported familiarity with selected ICD‐related concepts and exploratory subgroup analyses by age, education, and professional experience. No missing data were observed, as all questionnaire items required a response.

Prior to dissemination, the questionnaire was reviewed by two cardiologists with expertise in implantable cardiac devices and two senior paramedics involved in academic teaching to assess face validity, clarity, and clinical relevance of the items. Then, a pilot survey was performed on 10 paramedics. Internal consistency was explored for a subset of three dichotomous items, referring to declared familiarity with selected ICD‐related concepts (Questions 2, 5, and 6). Given the descriptive purpose of the study and the heterogeneous nature of the items—addressing distinct aspects such as terminology, ECG features, and deactivation procedures—the questionnaire was not designed as a psychometric scale measuring a single latent construct. Therefore, internal consistency was assessed only as an ancillary metric. The resulting Cronbach’s *α* was low (0.47), indicating limited internal consistency of the selected items. This limitation further restricts the interpretability of the results and supports treating the instrument strictly as a descriptive survey tool rather than a validated scale for assessing knowledge or competence.

In the first section, participants provided demographic and professional background information, including gender, age, education level, city size of employment, and work experience. Next, respondents were asked whether they had ever assisted a patient experiencing an ICD discharge. They were then asked whether they were familiar with the term “inappropriate ICD discharge.” To ensure a standardized understanding, a definition of inappropriate ICD discharge was presented before they proceeded to the next section. The subsequent part of the survey focused on paramedics’ experiences with [1] inappropriate ICD discharges [2] and ICD discharges during cardiopulmonary resuscitation (CPR). Participants were also asked whether they knew how to deactivate high‐energy ICD therapy and if they could recognize pacemaker activity on an ECG. In addition, the survey inquired whether their ambulance was equipped with a magnet for deactivating high‐energy ICD therapy, and whether this magnet was a commercially available product or an improvised tool. A section of the questionnaire addressing self‐reported experience and declared familiarity with ICDs is presented in Table 1.

**TABLE 1:** A section of the questionnaire addressing self‐reported experience and declared familiarity with implantable cardioverter‐defibrillators.

Question	Number
Have you provided assistance to a person experiencing a discharge of an implantable cardioverter‐defibrillator in your emergency practice?	1
Do you know what the term “inappropriate discharge” (“inappropriate intervention”) means in the context of the work of an implantable cardioverter‐defibrillator?	2
Have you provided assistance to a person experiencing an inappropriate discharge of an implantable cardioverter‐defibrillator in your emergency practice?	3
Have you provided assistance to a person experiencing a discharge of an implantable cardioverter‐defibrillator during cardiopulmonary resuscitation?	4
Do you know how to stop defibrillator discharges in case of inappropriate interventions?	5
Do you know how to identify proper pacemaker function on an ECG recording?	6

### 2.4. Sample Size Calculation

At the time the study began, the exact number of paramedics in Poland was unknown. The lack of precise data is primarily due to the absence of a professional registry maintained by a reliable authority. Not all individuals who complete studies in paramedic science pursue work in ambulances. Based on media opinions within the paramedic community, this number is estimated to be around 12,000. Therefore, this value was used by the authors for further calculations. The sample size calculation was approximate and exploratory, intended to guide recruitment rather than ensure statistical power or precise population estimates. Assuming a confidence level of 95% with a maximum margin of error of 5%, the estimated sample size for the study was 375 individuals.

### 2.5. Statistical Analysis

Analysis was performed using the Statistica 12 software (TIBCO Inc., Tulsa, OK, USA). Descriptive statistics of measurable variables were performed. The categorical variables were expressed as numbers (*n*) with percentages (%). Quantitative data were first checked for normal distribution using the Shapiro–Wilk test. As they did not present a normal distribution, they were presented as median (interquartile range). To assess statistical dependencies between qualitative variables, the chi‐square test was used. To evaluate the statistical significance of differences in quantitative variables, the Mann–Whitney test was applied. Given the descriptive aim, nonprobability sampling, and self‐reported nature of the data, multivariable modeling was not performed, and observed associations should not be interpreted as independent effects. A value of *p* < 0.05 was considered statistically significant.

## 3. Results

### 3.1. Study Group

A total of 340 paramedics completed the survey. Because recruitment was based on an open‐link design (email distribution and a QR code at a national conference), a precise response rate could not be calculated. The majority of participants were male (78.24%, *n* = 266), while females accounted for 21.76% (*n* = 74). The median age of respondents was 33 years (IQR: 28–40), with ages ranging from 22 to 52 years. The median work experience was 8 years (IQR: 4–16), with a minimum of 1 year and a maximum of 30 years.

Only 27 respondents (7.93%) reported having access to a magnet needed for ICD deactivation in their ambulance. Among these, 23 cases involved the use of a self‐prepared improvised magnet rather than a commercially available one, which was available only in 4 cases (1.12%). The detailed characteristics of the study group are presented in Table 2.

**TABLE 2:** Characteristics of the study group.

Variable	Value^∗^
Age	33 [28–40]

*Sex*	
Male	78.24 (266)
Female	21.76 (74)

*Education level*	
Bachelor’s degree (1st level)	68.24 (232)
Master’s degree (2nd level)	19.71 (67)
Postsecondary education	12.06 (41)

*Workplace location*	
City with up to 50 k residents	7.94 (27)
City over 500 k residents	33.53 (114)
City with 50 k–150 k residents	20.88 (71)
Village	23.53 (80)
City with 150 k–500 k residents	14.12 (48)
Work experience (years)	8 [4–16]
Is there a magnet dedicated to deactivating ICDs in the ambulance you work in?	7.93 (27)

^∗^Values were presented an % (*n*) or median (IQR).

### 3.2. Answers for Questions

The majority of respondents (91%, *n* = 310) reported having assisted a patient experiencing an ICD discharge during their practice. Notably, individuals with lower educational levels were more likely to report such experiences, and this difference was statistically significant (*p* = 0.01).

Fifty‐three percent of paramedics (*n* = 180) reported assisting a patient experiencing an inappropriate discharge. Within this group, respondents with higher education were significantly more likely to provide a positive response (*p* = 0.01).

Thirty‐five percent (*n* = 121) of respondents reported exposure to a patient experiencing an ICD discharge during CPR.

Eighty‐three percent (*n* = 282) of respondents reported familiarity with the term “inappropriate ICD discharge,” and 84% (*n* = 285) declared familiarity with ECG features associated with ICDs. In addition, 61% (*n* = 207) reported being familiar with methods for terminating inappropriate ICD discharges.

Respondents with higher education levels reported greater self‐declared familiarity with the analyzed issues, but these differences were not statistically significant.

A detailed breakdown of responses, including gender and educational level comparisons, is presented in Table 3.

**TABLE 3:** A detailed summary of responses to individual questions, including breakdowns by gender and educational level of respondents.

Question number	Total “yes”	Male	Female	*p* value	Postsecondary	Bachelor’s degree	Master’s degree	*p* value
1	91 (310)	92.86 (247)	85.14 (63)	0.03	100.00 (41)	91.81 (213)	83.58 (56)	0.01
2	84 (285)	83.83 (223)	83.78 (62)	0.99	80.49 (33)	83.19 (193)	88.06 (59)	0.52
3	53 (180)	51.13 (136)	59.46 (44)	0.20	43.90 (18)	50.00 (116)	68.66 (46)	0.01
4	35 (121)	39.85 (106)	20.27 (15)	< 0.05	31.71 (13)	34.05 (79)	43.28 (29)	0.32
5	61 (207)	60.53 (161)	62.16 (46)	0.79	60.98 (25)	59.48 (138)	65.67 (44)	0.66
6	83 (282)	83.46 (222)	81.08 (60)	0.63	79.10 (53)	82.76 (192)	90.24 (37)	0.32

*Note:* Data were presented as % (*n*).

As expected, respondents with longer professional experience more frequently reported prior exposure to ICD discharges (median 10 vs. 4 years; *p* < 0.05), reflecting cumulative clinical exposure over time. No other consistent associations were observed. No statistically significant differences were identified for other analyzed variables, including gender and educational level. Subgroup analyses were exploratory and should not be interpreted as indicating causal relationships.

## 4. Discussion

The literature on paramedics’ preparedness for ICD management in prehospital settings is limited [12]. Our findings highlight two main observations: frequent declared exposure to ICD‐related clinical scenarios and limited reported access to ICD deactivation magnets in ambulances.

An ICD has four main functions: detecting atrial and ventricular electrical signals, identifying ventricular arrhythmias, terminating them via shock or antitachycardia pacing, and pacing for bradycardia control [13, 14]. An inappropriate shock occurs in about 30% of all ICD shocks and affects 10% of ICD patients. It is triggered by rhythms other than VT or VF, often due to supraventricular arrhythmias. Other causes include electromagnetic interference (e.g., MRI) and inappropriate detection (e.g., excessive T‐wave sensitivity) [14, 15]. Reports of ICD shocks during chest compressions indicate that such scenarios occur in practice. Inappropriate shocks increase the risk of anxiety, depression, reduced quality of life, myocardial damage, and mortality but do not cause significant harm to bystanders [5, 11, 16, 17]. A phantom shock is a perceived but nonexistent shock, often experienced by patients with prior traumatic ICD shocks, particularly those with anxiety or depression [18]. ES refers to three or more VT/VF episodes within 24 h requiring ICD therapy. Affecting ∼10% of ICD patients, ES is a strong predictor of poor outcomes [19, 20].

A large majority of respondents reported having assisted a patient experiencing an ICD discharge during their emergency practice, indicating that encounters with ICDs are not rare events for paramedics. Furthermore, more than one‐third of respondents reported having encountered an ICD discharge during CPR, highlighting that such scenarios may arise even in high‐acuity resuscitation settings.

Only 61% of respondents reported familiarity with methods for terminating inappropriate ICD discharges, and 83% declared familiarity with ECG features associated with pacemaker or ICD function. Similar variability in survey responses has been described in studies conducted among nurses [12, 21–23]. Importantly, our findings reflect self‐reported familiarity rather than objectively verified knowledge or practical skills and should be interpreted accordingly.

In this context, the proportion of paramedics in the present survey who reported familiarity with the term “inappropriate discharge” (83%) appears broadly comparable to findings reported in selected nursing cohorts, such as the 86.48% reported by Steffes et al. [21]. However, differences in professional roles, workplace settings, and survey design limit direct comparison. The abovementioned differences may stem from question design and workplace environment—Norwegian nurses worked in various hospital wards, while American participants were cardiology nurses from academic centers [21, 23].

Subgroup analyses indicated that respondents with greater age and longer professional practice more frequently reported prior exposure to ICD‐related emergencies. This finding is not unexpected and likely reflects cumulative clinical exposure over time rather than differences in inherent competence. In addition, respondents with lower levels of formal education less frequently reported self‐declared familiarity with selected ICD‐related concepts. This result may help identify groups that could particularly benefit from targeted educational initiatives.

Previous studies have reported inconsistent associations between education, experience, and ICD‐related survey performance [12, 22]. Higher scores were linked to prior ICD exposure, Advanced Cardiovascular Life Support certification, and electrophysiology unit work, whereas hospital‐based nurses more frequently demonstrated lower technical performance [21].

One of the most striking findings of this survey is the very limited reported availability of ICD deactivation magnets in ambulances. Current literature lacks guidelines on whether ambulances should be equipped with ICD‐deactivating magnets [9, 10]. In Poland’s Upper Silesia region, 1.7% of out‐of‐hospital cardiac arrest patients had an ICD or CRT‐D [24]. Current emergency medical service equipment standards in many countries, including Poland, do not explicitly require ICD deactivation magnets as mandatory equipment. However, this aspect was not directly assessed in the present study and should be interpreted cautiously.

The findings of the present survey can be interpreted in the context of the European Resuscitation Council Advanced Life Support guidelines, which provide specific recommendations regarding the management of patients with ICDs in emergency settings [11]. According to these guidelines, the use of a magnet is an accepted and reversible method to suspend ICD shock therapy in selected clinical scenarios, including inappropriate shocks, ES, and recurrent shocks in hemodynamically stable patients.

In this context, the limited reported availability of ICD deactivation magnets observed in our study may have practical implications for guideline adherence in prehospital care. While the ERC guidelines do not explicitly mandate the presence of magnets in ambulances, they assume timely access to such devices when indicated. The finding suggests potential variability between guideline recommendations and real‐world resource availability.

From a practical perspective, these findings may be relevant in the context of prehospital advanced life support decision‐making, particularly in scenarios such as recurrent ICD shocks, suspected ES, or cardiac arrest requiring cardiopulmonary resuscitation. In such situations, paramedics may face decisions regarding the potential use of a magnet to temporarily suspend ICD therapy, appropriate pad placement to avoid device interference, or differentiation between appropriate and inappropriate shocks in a dynamic clinical setting.

In this context, several potential approaches may be considered, although these remain hypothesis‐generating and are not directly supported by the present data. For example, these might include the availability of ICD deactivation magnets in ambulances, incorporation of practical guidance on their use into paramedic training, or the development of local protocols for prehospital application. Similar observations have been reported in other healthcare settings [21–23].

Overall, the limited availability of ICD deactivation magnets observed in this survey should be interpreted as a descriptive signal of potential variability in equipment provision, rather than as evidence of individual practitioner inadequacy. Importantly, such measures would require evaluation in future studies to assess their feasibility, safety, and impact on clinical practice.

### 4.1. Limitations

This study has important limitations. First, the use of convenience sampling and recruitment during a scientific congress likely resulted in selection bias toward more professionally engaged paramedics, which could lead to overestimation of reported exposure and familiarity. Second, the survey relied on self‐reported data rather than objective testing of skills, which may not reflect actual clinical competence. Third, the questionnaire was developed by the authors and was not formally validated as a psychometric instrument. The items were intentionally designed to be brief and predominantly dichotomous, focusing on capturing declared familiarity and self‐reported exposure rather than objectively verified knowledge, skills, or clinical competence. In addition, the observed low internal consistency (Cronbach’s *α* = 0.47) indicates limited coherence among the included items and further supports interpreting the questionnaire as a descriptive survey tool rather than a scale intended to measure a single underlying construct. Consequently, the findings should be understood as reflecting self‐reported perceptions and experiences, not as an objective assessment of knowledge, preparedness, or clinical proficiency, and clearly separated from any implication about actual preparedness or competence. Accordingly, any system‐level implications discussed in this study should be considered hypothesis‐generating only and not directly supported by the collected data.

As this study was conducted using an online survey format, several limitations inherent to internet‐based research should be acknowledged. Some factors may affect the generalizability of the findings. Participation was voluntary and limited to individuals with access to electronic devices and internet connectivity, which may have influenced the composition of the sample. The recruitment strategy may have underrepresented paramedics without access to online platforms or those not participating in academic events, potentially biasing the sample toward urban or more engaged EMS settings. The open‐link design did not allow for individual tracking of invitations or calculation of a precise response rate, and duplicate participation could not be entirely excluded. Although duplicate entries could not be formally excluded due to the open‐link design, their impact is likely limited given the professional context of the survey and the lack of incentives for repeated participation.

Despite these limitations, this study identifies areas that may warrant further investigation. Future research could incorporate validated assessment tools, objective knowledge testing, or observational audits of ambulance equipment to better characterize prehospital preparedness for ICD‐related emergencies. Interventional studies evaluating targeted educational initiatives or standardized equipment provision may also be valuable.

## 5. Conclusions

This descriptive survey indicates that Polish paramedics commonly report encounters with ICD‐related situations in prehospital settings. Reported access to ICD deactivation magnets was rare. Respondents also reported variable familiarity with selected ICD‐related terms and procedures; however, these data reflect self‐reports and should not be interpreted as an objective assessment of knowledge or competence. Limited magnet availability may reflect system‐level variability in equipment provision relevant to guideline implementation. Further research using validated tools and objective measures is warranted.

**Nomenclature**
CPR: Cardiopulmonary resuscitation
ES: Electrical storm
ICDs: Implantable cardioverter‐defibrillators
VF: Ventricular fibrillation
VT: Ventricular tachycardia

## Author Contributions

Supervision and project administration: A.B. Funding acquisition: A.B. and T.K. Conceptualization and methodology: W.T. and T.K. Data collection: T.K. and R.W. Data analysis and interpretation: W.T., T.K., M.D., M.S., M.K., and R.W. Original manuscript drafting: W.T., T.K., M.D., M.S., M.K., and R.W. Manuscript review and editing: W.T., T.K., and A.B. Visualization and preparation of figures and tables: M.D., M.S., M.K., and R.W. Team coordination: W.T. and T.K.

## Funding

This work received no specific grant from any funding agency in the public, commercial, or not‐for‐profit sectors. Publication‐related costs were covered by the statutory funds of the University.

## Disclosure

All authors approved the final version of the manuscript.

## Ethics Statement

According to Polish law and the principles of Good Clinical Practice, survey studies do not meet the criteria of a medical experiment. Therefore, obtaining approval from the bioethics committee is generally not required.

## Consent

Informed consent was implied by voluntary participation in the anonymous survey.

## Conflicts of Interest

The authors declare no conflicts of interest.

## Supporting Information

Additional supporting information can be found online in the Supporting Information section.

## Supporting information


**Supporting Information** The supporting information include the reporting checklists for CONSORT and STROBE guidelines. These checklists have been provided to ensure transparency and completeness in the reporting of the study. They contain detailed information regarding the study design, methodology, data collection procedures, and analytical approaches applied in this research. Their inclusion allows readers and reviewers to assess the methodological rigor and reproducibility of the study in accordance with established reporting standards.

## Data Availability

The data that support the findings of this study are available from the corresponding author upon reasonable request.
