# Supplementary material for: ICD‐Related Emergencies in Prehospital Care: A Descriptive Survey of Self‐Reported Experience and Equipment Availability Among Polish Paramedics
Source: Emerg Med Int. 2026 May 28;2026:2737258. doi: 10.1155/emmi/2737258 (PMC13217388; doi:10.1155/emmi/2737258)
Supplement: Supplementary file 1 — Supporting Information The supporting information include the reporting checklists for CONSORT and STROBE guidelines. These checklists have been provided to ensure transparency and completeness in the reporting of the study. They contain detailed information regarding the study design, methodology, data collection procedures, and analytical approaches applied in this research. Their inclusion allows readers and reviewers to assess the methodological rigor and reproducibility of the study in accordance with established reporting standards. [file EMMI-2026-2737258-s001.zip › Supplementary Material 1 - STROBE Checklist.docx]

|  | **Recommendation** | **Manuscript section** |
| --- | --- | --- |
| 1 | Indicate the study’s design in the title or abstract | Title; Abstract – Methods |
| 2 | Provide an informative and balanced summary | Abstract |
| 3 | Explain the scientific background and rationale | Introduction |
| 4 | State specific objectives | Introduction |
| 5 | Present key elements of study design early | Methods – Study protocol |
| 6 | Describe the setting, locations, and dates | Methods – Study protocol |
| 7 | Describe eligibility criteria and selection | Methods – Study protocol and recruitment |
| 8 | Describe how variables were measured | Methods – Study protocol |
| 9 | Describe efforts to address bias | Methods – Study protocol; Limitations |
| 10 | Explain how study size was arrived at | Methods – Sample size calculation |
| 11 | Explain handling of quantitative variables | Methods – Statistical analysis |
| 12a | Describe statistical methods | Methods – Statistical analysis |
| 12b | Describe methods for subgroup analyses | Methods – Statistical analysis |
| 12c | Explain handling of missing data | Methods – Outcomes and Questionnaire |
| 13 | Describe numbers of participants at each stage | Results – Study group |
| 14 | Give characteristics of study participants | Results – Study group; Table 2 |
| 15 | Report numbers of outcome events | Results – Answers for questions |
| 16 | Report main results | Results |
| 17 | Report subgroup analyses | Results – Answers for questions |
| 18 | Summarize key results | Discussion |
| 19 | Discuss limitations | Discussion – Limitations |
| 20 | Provide cautious interpretation | Discussion |
| 21 | Discuss generalisability | Discussion; Limitations |
| 22 | Give funding source | Funding |

Supplementary Material 1 – STROBE Checklist
