# Supplementary material for: ICD‐Related Emergencies in Prehospital Care: A Descriptive Survey of Self‐Reported Experience and Equipment Availability Among Polish Paramedics
Source: Emerg Med Int. 2026 May 28;2026:2737258. doi: 10.1155/emmi/2737258 (PMC13217388; doi:10.1155/emmi/2737258)
Supplement: Supplementary file 1 — Supporting Information The supporting information include the reporting checklists for CONSORT and STROBE guidelines. These checklists have been provided to ensure transparency and completeness in the reporting of the study. They contain detailed information regarding the study design, methodology, data collection procedures, and analytical approaches applied in this research. Their inclusion allows readers and reviewers to assess the methodological rigor and reproducibility of the study in accordance with established reporting standards. [file EMMI-2026-2737258-s001.zip › Supplementary Material 2 - CROSS Checklist.docx]

| **Item** | **Recommendation** | **Manuscript section** |
| --- | --- | --- |
| 1 | Describe survey design | Methods – Study protocol |
| 2 | Ethical approval and consent | Methods – Legal Issues; |
| 3 | Explain data protection and GDPR compliance | Methods – Legal Issues |
| 4 | Describe recruitment process | Methods – Study protocol and recruitment |
| 5 | Describe sampling strategy | Methods – Study protocol and recruitment, Sample size calculation |
| 6 | Describe survey administration mode | Methods – Study protocol |
| 7 | Explain open vs closed survey | Methods – Study protocol and recruitment, Outcomes and Questionnaire |
| 8 | Describe prevention of multiple entries | Methods – Study protocol and recruitment |
| 9 | Describe questionnaire development | Methods – Study protocol and recruitment |
| 10 | Report pretesting / pilot testing | Methods – Study protocol and recruitment |
| 11 | Describe completeness checks | Methods – Statistical analysis |
| 12 | Report response rate calculation | Methods – Outcomes and Questionnaire |
| 13 | Describe handling of incomplete questionnaires | Methods – Statistical analysis |
| 14 | Report data analysis methods | Methods – Statistical analysis |
| 15 | Report limitations of online surveys | Discussion – Limitations |

Supplementary Material 2 - CROSS Checklist
